# Monophyletic blowflies revealed by phylogenomics

**DOI:** 10.1186/s12915-021-01156-4

**Published:** 2021-10-27

**Authors:** Liping Yan, Thomas Pape, Karen Meusemann, Sujatha Narayanan Kutty, Rudolf Meier, Keith M. Bayless, Dong Zhang

**Affiliations:** 1 School of Ecology and Nature Conservation, Beijing Forestry University, Beijing, China; 2 Natural History Museum of Denmark, University of Copenhagen, Copenhagen, Denmark; 3 Evolutionary Biology & Ecology, University of Freiburg, Freiburg, Germany; 4 Zoologisches Forschungsmuseum Alexander Koenig (ZFMK)/Zentrum für Molekulare Biodiversitätsforschung (ZMB), Bonn, Germany; 5 Australian National Insect Collection, CSIRO National Research Collections Australia (NRCA), Canberra, Australia; 6 Department of Biological Sciences, National University of Singapore, Singapore, Singapore; 7 Tropical Marine Science Institute, National University of Singapore, Singapore, Singapore; 8 Museum für Naturkunde, Leibniz Institute for Evolution and Biodiversity Science, Center for Integrative Biodiversity Discovery, Berlin, Germany; 9 Department of Entomology, California Academy of Sciences, San Francisco, USA

**Keywords:** Calyptratae, Transcriptome, Genome, Phylogeny, Coloration

## Abstract

**Background:**

Blowflies are ubiquitous insects, often shiny and metallic, and the larvae of many species provide important ecosystem services (e.g., recycling carrion) and are used in forensics and debridement therapy. Yet, the taxon has repeatedly been recovered to be para- or polyphyletic, and the lack of a well-corroborated phylogeny has prevented a robust classification.

**Results:**

We here resolve the relationships between the different blowfly subclades by including all recognized subfamilies in a phylogenomic analysis using 2221 single-copy nuclear protein-coding genes of Diptera. Maximum likelihood (ML), maximum parsimony (MP), and coalescent-based phylogeny reconstructions all support the same relationships for the full data set. Based on this backbone phylogeny, blowflies are redefined as the most inclusive monophylum within the superfamily Oestroidea not containing Mesembrinellidae, Mystacinobiidae, Oestridae, Polleniidae, Sarcophagidae, Tachinidae, and Ulurumyiidae. The constituent subfamilies are re-classified as Ameniinae (including the Helicoboscinae, **syn. nov.**), Bengaliinae, Calliphorinae (including Aphyssurinae, **syn. nov.**, Melanomyinae, **syn. nov.**, and Toxotarsinae, **syn. nov.**), Chrysomyinae, Luciliinae, Phumosiinae, Rhiniinae **stat. rev.**, and Rhinophorinae **stat. rev**. Metallic coloration in the adult is shown to be widespread but does not emerge as the most likely ground plan feature.

**Conclusions:**

Our study provides the first phylogeny of oestroid calyptrates including all blowfly subfamilies. This allows settling a long-lasting controversy in Diptera by redefining blowflies as a well-supported monophylum, and blowfly classification is adjusted accordingly. The archetypical blowfly trait of carrion-feeding maggots most likely evolved twice, and the metallic color may not belong to the blowfly ground plan.

**Supplementary Information:**

The online version contains supplementary material available at 10.1186/s12915-021-01156-4.

“these summerflies have blown me full of maggot ostentation.”

William Shakespeare [1] *Love’s Labour’s Lost*

## Background

**Fig. 1:**
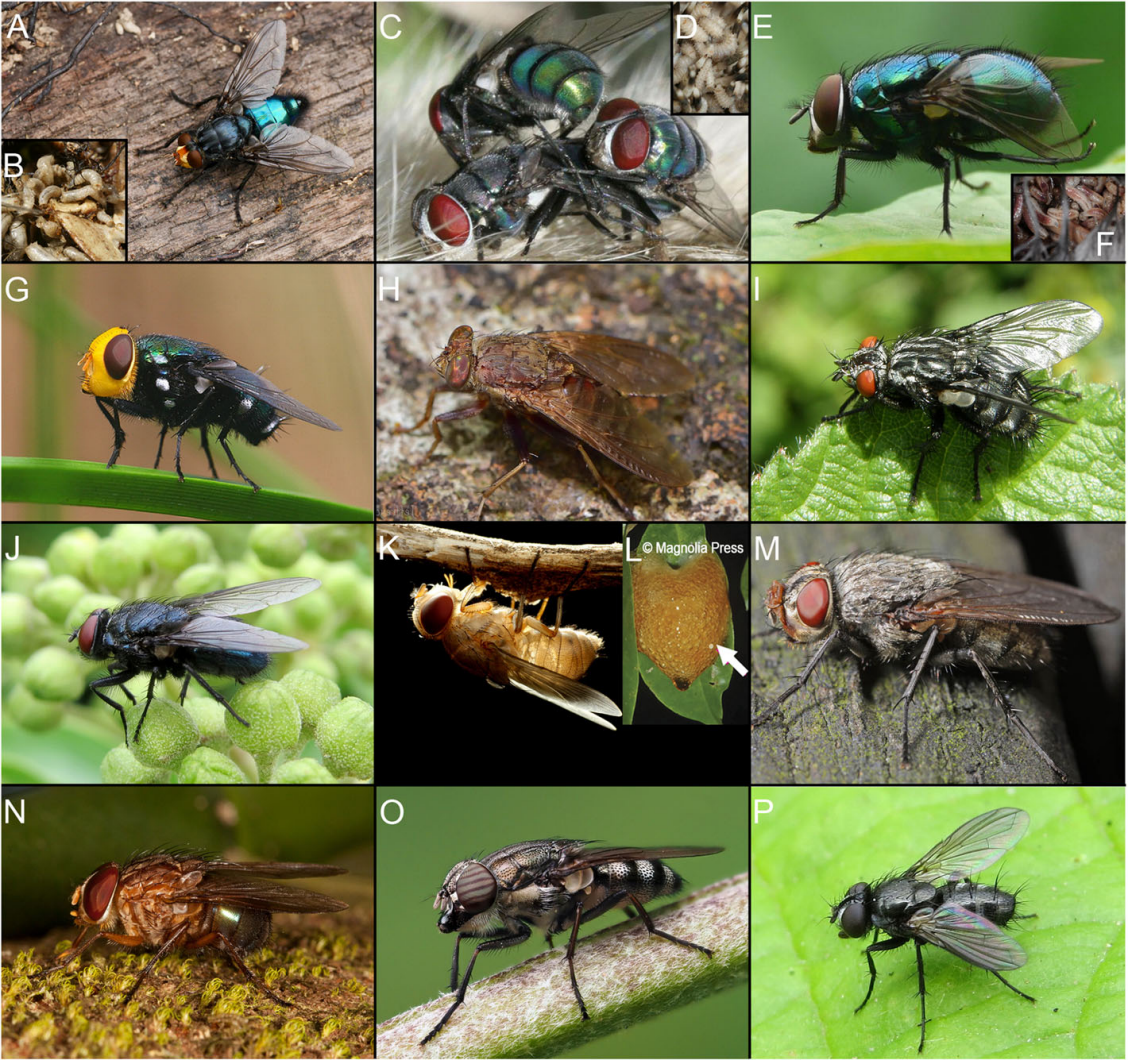
Representative taxa of calliphorids, Mesembrinellidae and Polleniidae. **A**, **B** Calliphorinae. **A** *Calliphora* sp. **B** *Calliphora* sp., larvae feeding on dead bird. **C**, **D** Chrysomyinae. **C** *Chrysomya* sp. **D** *Chrysomya albiceps*, larvae feeding on dead hedgehog. **E**, **F** Luciliinae. **E** *Lucilia* sp. **F** *Lucilia* sp., larvae feeding on dead bird. **G** Ameniinae (*Amenia* sp.). **H** Bengaliinae (*Bengalia* sp.). **I** Helicoboscinae (*Eurychaeta palpalis*). **J** Melanomyinae (*Melinda viridicyanea*). **K**, **L** Phumosiinae. **K** *Caiusa* sp. **L** – *Caiusa* sp., egg on foam mass of the shrub frog *Chiromantis nongkhorensis* [5] (reproduced with permission from copyright holder). **M** Polleniidae (*Pollenia* sp.). **N** Mesembrinellidae (*Mesembrinella* sp.). **O** Rhiniidae (*Stomorhina lunata*). **P** Rhinophoridae (*Rhinophora lepida*). **A**, **B**, **G**, **H**, **I**, **J**, **O**, and **P** are from Flickr; **C**, **D**, **E**, **F**, and **M** are from Diptera.info; **K** is from antroom

**Fig. 2:**
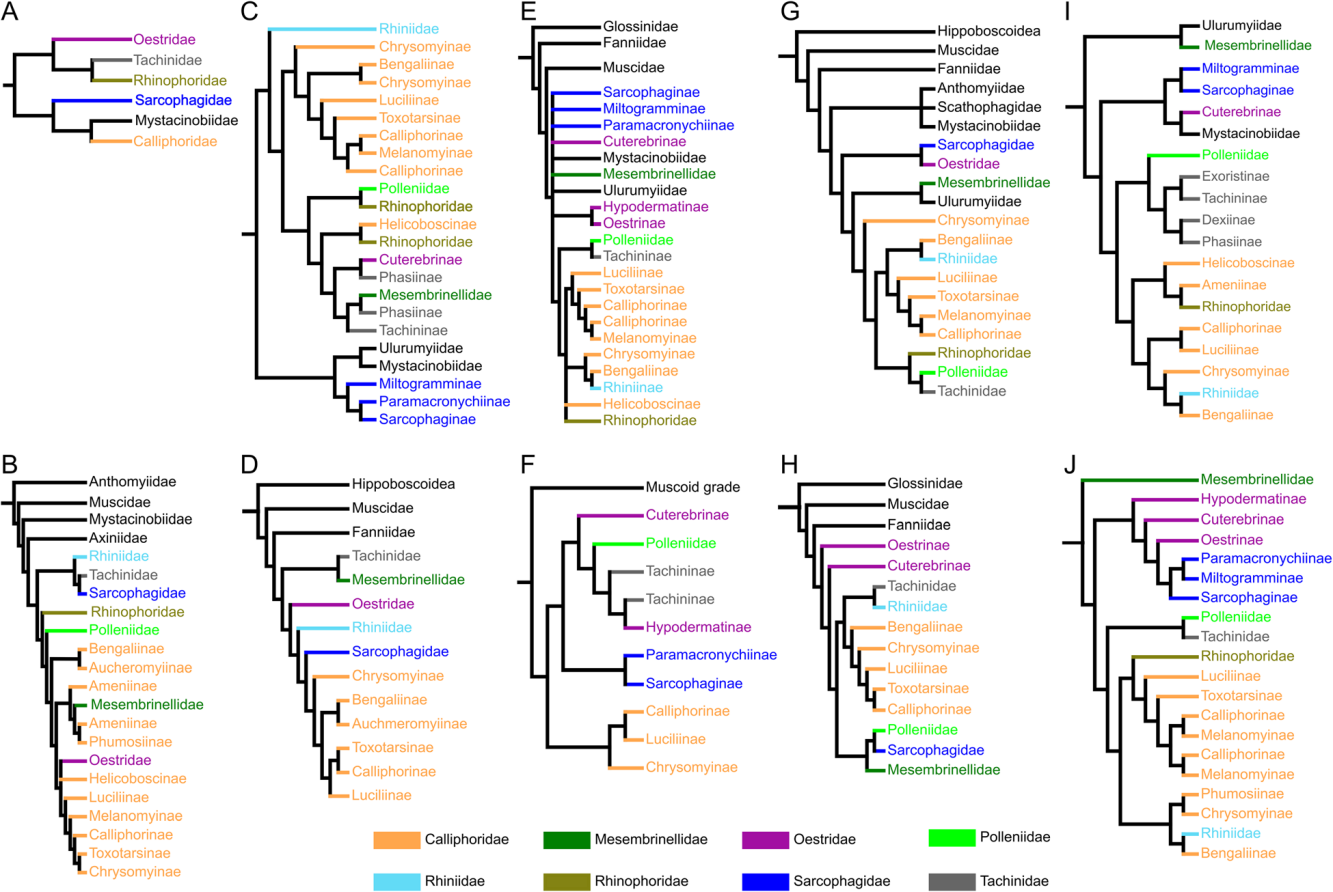
Phylogeny of Oestroidea in previous studies. **A** McAlpine [11] (morphology). **B** Rognes [12] (morphology). **C** Kutty et al. [13] (combination of mitochondrial and nuclear genes). **D** Marinho et al. [14] (combination of mitochondrial and nuclear genes). **E** Singh & Wells [15] (combination of mitochondrial and nuclear genes). **F** Zhang et al. [16] (mitogenomic data). **G** Cerretti et al. [17] (combination of mitochondrial and nuclear genes). **H** Marinho et al. [18] (combination of mitochondrial and nuclear genes). **I** Kutty et al. [19] (phylotranscriptomic data). **J** Buenaventura et al. [20] (ultra-conserved elements)

Blowflies (Diptera: Calyptratae, Calliphoridae) are among the most familiar insects to humans [2–4]. They are abundant on all continents except Antarctica, and the anthropophilic species are well known for their association with carrion and decaying food (Fig. 1) [6–8]. Many species have distinctive metallic coloration, and the family name Calliphoridae means “beauty bearer” in Greek [6], alluding to the beautiful shiny blue, green, or copper iridescence of the adult flies. Blowflies are also the first insects recognized in writing, as some cuneiform clay tablets mention these flies more than 3600 years ago [6]. Some species, e.g., *Cochliomyia hominivorax*, are infamous for causing significant economic losses to livestock because their maggots invade healthy tissue [9]. The larvae of many species of blowflies catalyze putrefaction and decay [10] and can be used in forensics to determine the time of death of corpses [10]. What is less broadly known, and in striking contrast to their cultural prominence, is that there is no consensus resolution as to which oestroid clade should be termed Calliphoridae, because the group has been repeatedly shown to be either para- or polyphyletic based on both molecular and morphological evidence (Fig. 2) [12, 13, 19, 21].

The family-group name Calliphoridae was erected for a large assemblage of calyptrate flies [22], but Girschner [23] was the first to narrow its definition when he restricted it to carrion-breeding oestroids, thus rendering the family a taxon of convenience for those oestroid flies that do not belong to, for example, the more easily-diagnosable flesh flies (Sarcophagidae), bot flies (Oestridae), or tachinid flies (Tachinidae). Indeed, McAlpine [11] and Pape [24] were the last authors to claim some evidence for calliphorid monophyly based on putative synapomorphies, but since then, both morphological and molecular evidence has pointed to the non-monophyly of calliphorids [12–15, 17]. This evidence was used to improve the definitions and circumscriptions of the constituent subfamilies [12, 25–27], but defining a monophyletic Calliphoridae had to be postponed until an analysis could be carried out that included sufficient data for representatives of all subfamilies and employed sophisticated analyses to resolve all critical relationships with confidence. Until now, calliphorids have been left as the last major assemblage in the Calyptratae [11–13, 19, 24] that is defined on “what it is not” [8, 15].

The taxonomic composition of blowflies has been controversial for decades, which is reflected in a large number of recognized subfamilies, with more than a dozen in widespread use: Ameniinae, Aphyssurinae, Auchmeromyiinae, Bengaliinae, Calliphorinae, Chrysomyinae, Helicoboscinae, Luciliinae, Melanomyinae, Mesembrinellinae, Polleniinae, Phumosiinae, Prosthetosomatinae, Rhiniinae, Rhinophorinae, and Toxotarsinae [7, 12, 26, 28–30]. Some of these were later raised to family rank: Mesembrinellidae, Polleniidae, Rhiniidae, and Rhinophoridae [13–15, 31–34], while others have been relegated into synonymy: Auchmeromyiinae (under Bengaliinae) and Prosthetosomatinae (under Rhiniidae) [5, 13, 14].

With the availability of phylogenomic data (e.g., [19, 35]) and advances in data analysis [36], it is now possible to address phylogenetic questions based on a phylogenetic signal from thousands of genes. Here, we perform a phylogenomic analysis of blowflies based on comprehensive taxon sampling of all recognized subfamilies, reconstruct the phylogenetic backbone of calliphorid subfamilies, to eventually propose the first rigorous definition of blowflies. Furthermore, we examine the robustness of our conclusions through the use of multiple ortholog reference sets and taxon subsampling.

## Results

### Phylogenomic data generation

Novel phylogenomic data are provided for nine species of blowflies, representing nine of 10 subfamilies (Additional file 1: Table S1). Details of assemblies and number of recovered orthologous genes for each species are presented in Table S1 (Additional file 1).

### Phylogeny reconstruction

**Fig. 3:**
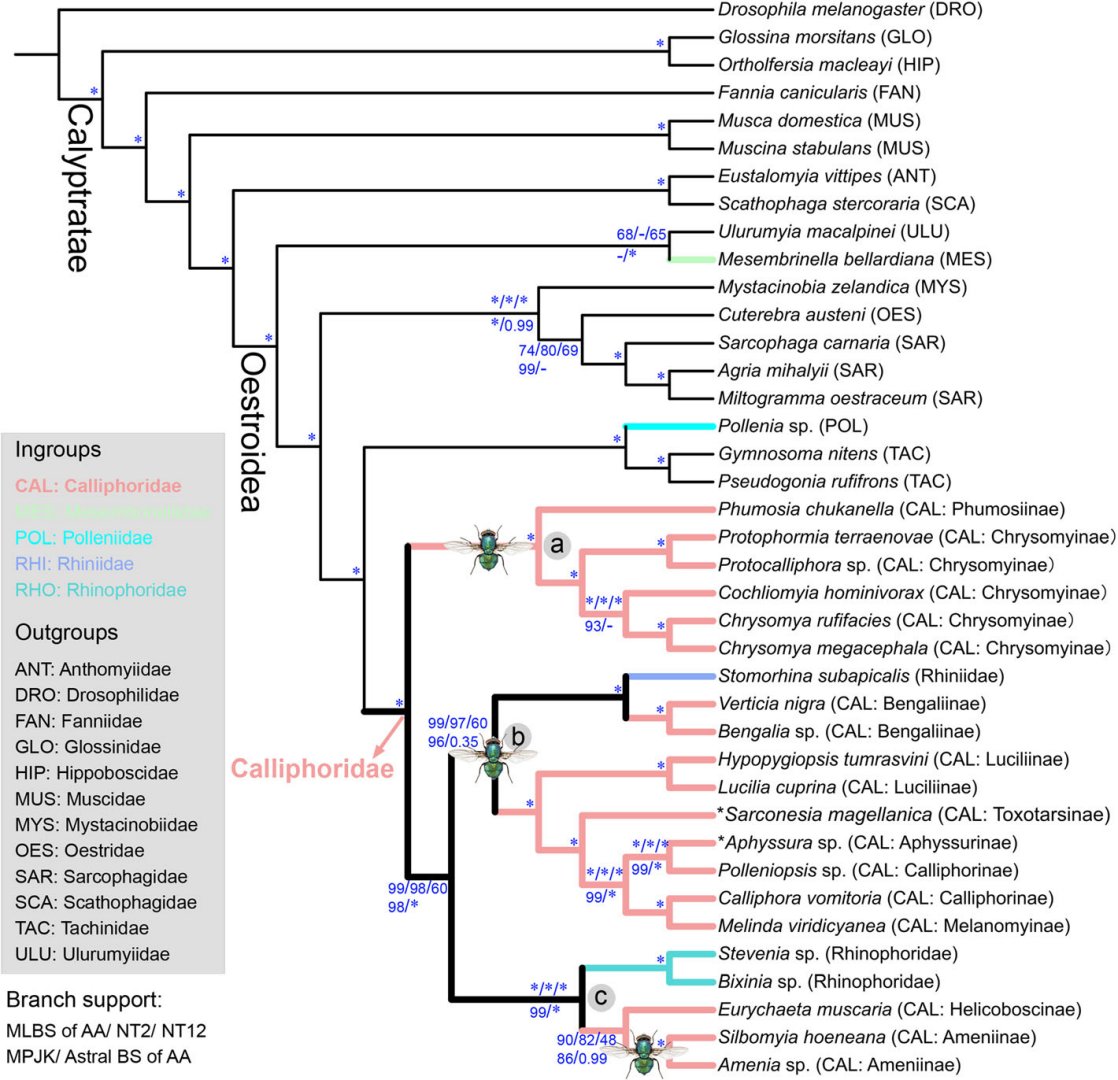
Maximum likelihood (ML) tree inferred from the amino acid matrix of dataset Dref_Ltax, with support values of ML bootstrap (MLBS), maximum parsimony jackknife (MPJK), and ASTRAL bootstrap (Astral BS) presented at nodes. The flies on the branches indicate origins of adult metallic color within calliphorids. The asterisk (*) and hyphen (-) at nodes indicate full support and branch not recovered, respectively. Species marked with asterisk (*) are sequenced with genomic data. Ingroup branches are colored according to family classification, as explained in the legend

**Fig. 4:**
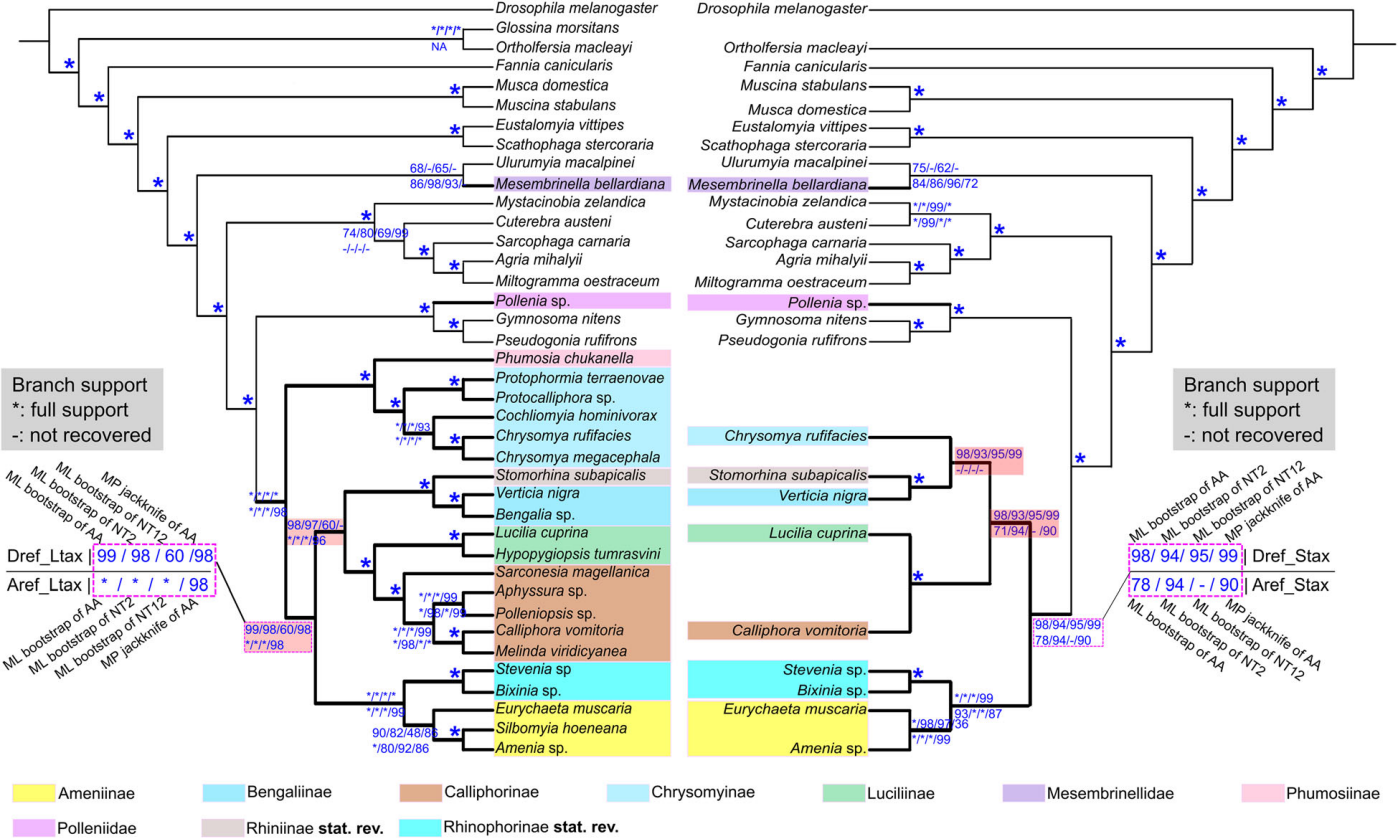
Phylogenetic topology compared between reconstructions based on datasets with larger (left) and smaller (right) taxon sampling. Numbers above nodes of the left cladogram are maximum likelihood (ML) bootstrap values of phylogeny inferred from the dataset Dref_Ltax of amino acid (AA), 2nd-codon positions (NT2), 1st & 2nd-codon position (NT12), and maximum parsimony jackknife value of phylogeny inferred from the dataset of amino acid (AA). Numbers below nodes of the left cladogram are ML bootstrap values of phylogeny inferred from dataset of AA, NT2, NT12, and MP jackknife value of phylogeny inferred from AA of dataset Aref_Ltax. Numbers above nodes of the right cladogram are ML bootstrap values of phylogeny inferred from dataset of AA, NT2, NT12, and MP jackknife value of phylogeny inferred from AA of dataset Dref_Stax. Numbers below nodes of the right cladogram are ML bootstrap values of phylogeny inferred from dataset of AA, NT2, NT12, and MP jackknife value of phylogeny inferred from AA of dataset Aref_Stax. The reddish boxes indicate nodes which conflict between analyses

Phylogenetic reconstructions using different matrices (Table 1) yielded similar topologies with only minor differences in the placement of Chrysomyinae and Mesembrinellidae (Figs 3 and 4; Additional file 2: Fig. S1). Calliphorids were recovered as monophyletic with the inclusion of the families Rhinophoridae and Rhiniidae. Calliphoridae are thereby redefined as the most inclusive group within the superfamily Oestroidea not containing Mesembrinellidae, Mystacinobiidae, Oestridae, Polleniidae, Sarcophagidae, Tachinidae, and Ulurumyiidae, and the family is here divided into three major clades (Fig. 3, clade a, b, and c). All traditionally recognized subfamilies were monophyletic except for the paraphyletic calliphorine grade, within which Aphyssurinae and Melanomyinae are nested (Figs. 3 and 4). The Neotropical Toxotarsinae are sister to the (Aphyssurinae-Calliphorinae-Melanomyinae), and this clade is sister to the monophyletic Luciliinae (Figs. 3 and 4). The clade ((Helicoboscinae, Ameniinae), Rhinophoridae) (clade c) emerged in all analyses with strong support, with Helicoboscinae (represented by *Eurychaeta muscaria*) invariably being the sister group to Ameniinae (Figs. 3 and 4). Chrysomyinae emerged as a sister group to Phumosiinae in a basal clade of calliphorids (clade a) in the reconstructions using datasets Dref_Ltax and Aref_Ltax (different reference taxa) with high support (Figs. 3 and 4), while the Chrysomyinae are placed as sister group to Luciliinae and Calliphorinae in reconstructions based on amino acid and second codon matrices of dataset Aref_Stax or to Bengaliinae and Rhiniidae in reconstructions based on dataset Dref_Stax (Fig. 4).

**Table 1:** Data set composition for the matrices used for phylogeny construction

Matrix	Treatment	Number of genes	Number of amino acids	Data completeness (Ca, Alistat)	Phylogenetic information (IC, MARE)
Dref_Ltax	Dataset generated with Diptera reference ortholog set and large taxon sampling	2221	1,190,119	0.709	0.61
Dref_Stax	Dataset generated with Diptera reference ortholog set and small taxon sampling	2003	1,014,045	0.717	0.60
Aref_Ltax	Dataset generated with the Antliophora reference ortholog set and large taxon sampling	1764	692,429	0.715	0.60
Aref_Stax	Dataset generated with the Antliophora reference ortholog set and small taxon sampling	1465	587,234	0.722	0.59

Polleniidae were consistently sister group to Tachinidae with full support (Figs. 3 and 4). The sister-group relationship between Mesembrinellidae and Ulurumyiidae was recovered in most analyses (but see Fig. 4). Occasionally, Mesembrinellidae were placed as a sister group to the non-Ulurumyiidae oestroids, but with low support (MP jackknife [MPJK] of Dref_Ltax: 98; MPJK of Dref_Stax: 100; MPJK of Aref_Ltax: 14; ML bootstrap [MLBS] of Dref_Ltax: 53; MLBS of Dref_Stax: 88).

#### Coalescent-based reconstruction

The coalescent approach yielded a subfamily-level topology similar to the concatenated ML tree based on the AA matrix of dataset Dref_Ltax (node support values in Fig. 3), differing in the relationship between Mystacinobiidae and Oestridae, which were recovered as sister groups in the coalescent phylogeny. Furthermore, *Cochliomyia hominivorax* was recovered as a sister group to *Chrysomya* in the concatenation-based phylogeny, while it is sister to all remaining chrysomyines in the coalescent-based phylogeny.

#### Phylogenetic position of Chrysomyinae

**Fig. 5:**
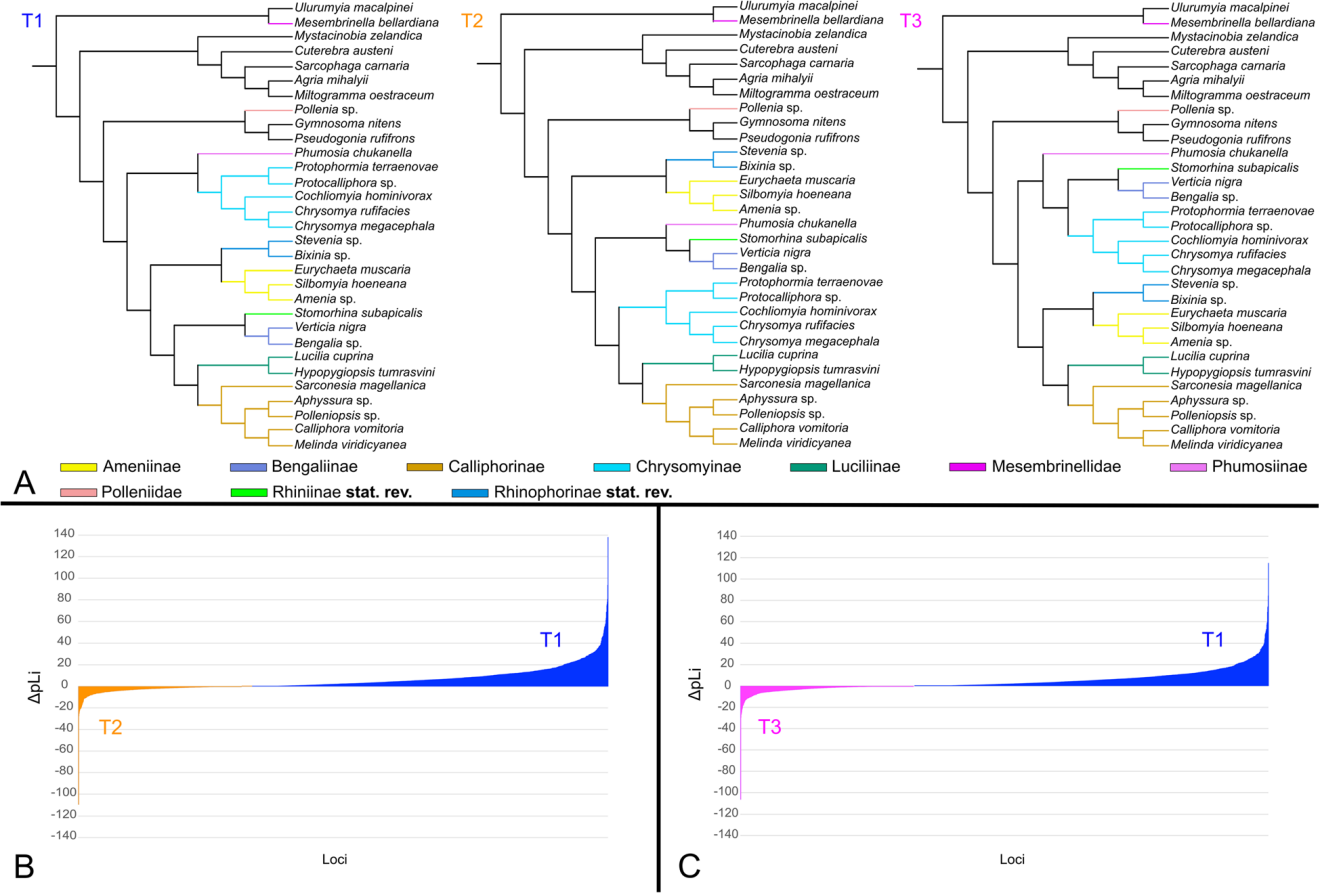
Results of partition log-likelihood analyses in terms of phylogenetic position of Chrysomyinae using amino acid alignments of dataset Dref_Ltax. **A** The three topologies. **B**, **C** Ranked distribution of ΔpLi of 2221 genes under the model estimated by IQ-TREE. **B** Genes favoring T1 (positive values) or T2 (negative values). **C** Genes favoring T1 (positive values) or T3 (negative values)

Within the calliphorids, only the placement of Chrysomyinae differed between reconstructions and data sets. In most analyses, the subfamily clusters with Phumosiinae and this combined clade is sister to the remaining Calliphoridae (T1 in Fig. 5A). This hypothesis is also favored in the remaining analyses although Chrysomyinae are sometimes placed as sister group to either Calliphorinae and Luciliinae (reconstructions based on amino acid and second codon matrices of dataset Aref_Stax) or Bengaliinae and Rhiniidae (all reconstructions based on matrices of dataset Dref_Stax). To test the fitness of data among phylogenies with different placements of Chrysomyinae, two additional ML trees were inferred using the amino acid matrix of the dataset with the most genes (i.e., Dref_Ltax, see Table 1), and constraining as monophyletic either Aphyssurinae-Calliphorinae-Melanomyinae-Toxotarsinae, Chrysomyinae, and Luciliinae) (T2 in Fig. 5A) or Chrysomyinae, Bengaliinae, and Rhiniidae (T3 in Fig. 5A). The likelihood score of each locus to the three hypotheses, T1, T2, and T3, were estimated (Additional file 3), and the score differences for each locus were recorded between T1 and T2 and between T1 and T3 (Fig. 5; Additional file 3). The results indicate that the hypothesis supported in most analyses (T1: see above) was favored by 1536 out of 2221 genes while T2 was only favored by 683 genes (Fig. 5B). T1 is also favored in a comparison with T3 (by 1489 instead of 727 genes, Fig. 5C). Furthermore, the AU test strongly supported T1 (Fig. 5A). The same hypothesis is supported by additional Kishino-Hasegawa, Shimodaira-Hasegawa, and AU tests for the AA matrix of dataset Dref_Ltax (*p*-KH = 1, *p*-SH = 1, *p*-AU = 1), although the AU test gave weak support for topology T2 (*p*-AU = 1.14e−08) and T3 (*p*-AU = 5.89e−41) (Table 2).

**Table 2:** Approximately unbiased (AU) topology tests under the model estimated by IQ-TREE

Tree	logL	*p*-KH^†^	*p*-SH^‡^	*p*-AU
T1	−13610170.38	1	1	1
T2	−13622645.67	0	0	1.14e−08
T3	−13620112.80	0	0	5.89e−41

^†^*p* value of one-sided Kishino-Hasegawa test [37]

^‡^*p* value of Shimodaira-Hasegawa test [38]

**Fig. 6:**
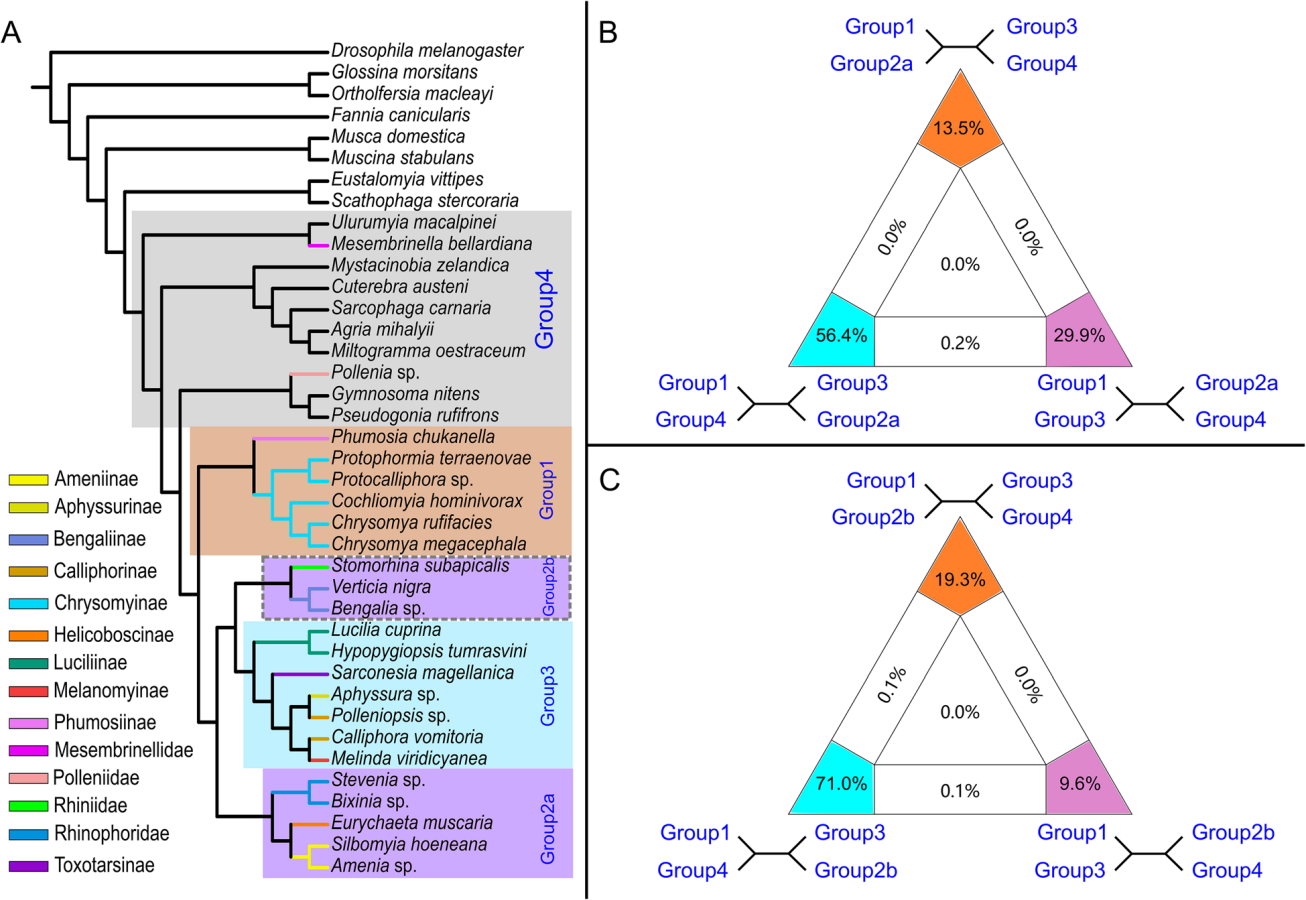
Four-cluster likelihood mapping (FcLM) of the phylogenetic position of Chrysomyinae using amino acid alignments of dataset Dref_Ltax. **A** Group definitions. **B**, **C** Two variations of FcLM based on concatenated amino acid alignments shown as 2D graphs, with phylogeny modified by excluding group 2b (**B**) or by excluding group 2a (**C**)

The modified FcLM revealed two different topologies depending on whether group 2b (Fig. 6A) or group 2a are pruned. Without group 2b (Fig. 6B), the support for Chrysomyinae and Phumosiinae (group 1) being sister group to Luciliinae, Aphyssurinae-Calliphorinae-Melanomyinae-Toxotarsinae (group 3) was 29.9%; i.e., lower than the support for placing Chrysomyinae and Phumosiinae (group 1) as the sister group to the remaining calliphorids (56.4%; Fig. 6B). When group 2a was excluded, the support for Chrysomyinae and Phumosiinae (group 1) as the sister group to the remaining calliphorids was 71.0% and thus far higher than the two competing hypotheses (Fig. 6C).

### Ancestral state reconstruction of adult metallic color

Metallic color has been considered as a ground plan character of blowflies [12]; however, the cuticle of the ancestral Calliphoridae was estimated to be non-metallic (probability = 65.81%; Additional file 2: Fig. S2; Additional file 4). Metallic color may have originated three times independently in the early evolution of Calliphoridae (Fig. 3): in the clade Chrysomyinae + Phumosiinae (clade a; probability = 96.37%) (Additional file 2: Fig. S2; Additional file 4), in the clade b ((Rhiniidae, Bengaliinae), (Luciliinae, Aphyssurinae-Calliphorinae-Melanomyinae-Toxotarsinae)) (probability = 47.43%), and in the Ameniinae (probability = 94.83%).

## Discussion

A rigorous definition of a monophyletic concept of the blowflies has been elusive because of incomplete taxon sampling at the subfamily level in previous studies [13–15, 19, 20]. Our study presents the first phylogenetic analyses including representatives of all blowfly subfamilies, with newly documented transcriptome and genomic data for nine species (Additional file 1: Table S1). This allows us to propose a monophyletic Calliphoridae and provide insight into several open questions relating to calyptrate phylogeny [12, 13, 19]. We here discuss the results based on the tree obtained based on analyses of amino acid alignments of the largest set of genes and taxa (Dref_Ltax: Fig. 3).

### Monophyletic Calliphoridae and blowfly classification

Based on this robustly supported phylogeny, we propose a broadly defined monophyletic Calliphoridae that excludes only the former calliphorid subfamilies Mesembrinellinae and Polleniinae, which is in line with recent proposals to treat these taxa as families [14, 27, 39]. The present broad definition of Calliphoridae is only one among multiple options for resolving calliphorid monophyly. Alternatively, a number of calliphorid subfamilies could be raised to family rank, as previously proposed for the Rhiniidae [13]. Arguments for bestowing a given rank to a particular clade can be drawn from various measures of morphological, biological, or phylogenetic distinctiveness, and tradition often weighs in. As discussed by Kallal et al. [40], the ranking may follow unspecified conventions of a research community, and reasoning for any given rank can be idiosyncratic and subjective. However, classifications matter as a framework for communication, and a reclassification of the calliphorids should be carefully crafted. Proposing a resolution to the paraphyly of Calliphoridae *sensu stricto* will promote stability in the long term. We favor a broad Calliphoridae, because it is close to the traditional calliphorids given that only the Mesembrinellidae and Polleniidae are excluded, and the Rhiniidae and Rhinophoridae are reclassified as subfamilies of Calliphoridae. Incidentally, the family group name Rhinophoridae dates from Robineau-Desviody [41], while the family group name Calliphoridae is younger [42], which means that an application should be submitted to the International Commission of Zoological Nomenclature for reversal of precedence.

The subfamily-level re-classification is proposed based on the backbone phylogeny of Calliphoridae (Table 3). We propose the Calliphorinae to include the former Aphyssurinae **syn. nov.**, Melanomyinae **syn. nov.**, and Toxotarsinae **syn. nov.** The clade consisting of Aphyssurinae, Melanomyinae, and Toxotarsinae is well supported and emerges in all our analyses (Figs. 3 and 4). Prior to our analysis, the placement of Aphyssurinae was unknown [28], but relationships between members of the other subfamilies in this clade have previously been analyzed [13, 15, 17] using a combination of mitochondrial and nuclear genes, but with a small taxon sample. The Calliphorinae and Melanomyinae emerged together in a recent phylogenomic study using protein-encoding ultraconserved elements (UCEs), although neither were monophyletic [20]. The position of Toxotarsinae within the re-defined, broader Calliphorinae has been corroborated by phylogenetic studies based on both Sanger and phylogenomic data [13, 20]. We therefore propose a redefined Calliphorinae sinking the former Aphyssurinae, Melanomyinae, and Toxotarsinae as subordinate taxa. This will have the added advantage of conserving the traditionally accepted sister-group relationship between Calliphorinae and Luciliinae. The former Helicoboscinae are proposed to be synonymized under Ameniinae, **syn. nov.**, thereby establishing a monophyletic subfamily containing rather robust, mostly macrolarviparous species feeding on live, dying, or dead snails [43, 44].

**Table 3:** Change of subfamily status within Calliphoridae proposed by the present study

Subfamilies and families of blowflies ***sensu lato***	Current status
Ameniinae	Valid
Aphyssurinae	Sunk into Calliphorinae
Bengaliinae	Valid
Calliphorinae	Valid
Chrysomyinae	Valid
Helicoboscinae	Sunk into Ameniinae
Luciliinae	Valid
Melanomyinae	Sunk into Calliphorinae
Mesembrinellidae	Valid family
Phumosiinae	Valid
Polleniidae	Valid family
Rhiniidae	Sunk into Calliphoridae as subfamily
Rhinophoridae	Sunk into Calliphoridae as subfamily
Toxotarsinae	Sunk into Calliphorinae

### Carrion breeding blowflies probably originated twice

All the carrion-breeding blowflies are found in the two clades (Calliphorinae *sensu lato* + Luciliinae) and Chrysomyinae. As these are separated by four nodes, carrion breeding in these two clades is most parsimoniously interpreted as having independent origins. This is surprising and challenges the traditional assumption that the common blowflies form a monophyletic group based on general appearance and life habits [12] (Fig. 1). In the present study, the tree topology that is overwhelmingly favored (Fig. 3, T1 in Fig. 5A) differs in some important respects from other trees (T2 and T3 in Fig. 5A) obtained using phylogenomic data. The hypothesis (T1) is well supported by partition log-likelihood analyses and AU test and is favored by the more sensitive FcLM analysis over the other two likely placements (Fig. 6). Interestingly, this placement of Chrysomyinae + Phumosiinae was also recovered in a coalescent-based phylogeny using transcriptome-derived ultraconserved elements [20], while it was rejected by a phylogenetic analysis based on concatenated genes [20].

### Is metallic color part of the blowfly ground plan?

Surprisingly, metallic adult cuticle, a long accepted ground plan character of blowflies, may best be considered as having evolved multiple times in the family. Blowflies are generally well-understood to be predominantly metallic [12]. However, the ancestor of blowflies may not have been a metallic fly, and it appears most likely that metallic colors evolved repeatedly during the early radiation of blowflies (Fig. 3).

## Materials and methods

### Specimen acquisition and taxon sampling

Blowfly specimens collected for RNA extraction were identified alive after capture. The male terminalia and/or a hind leg were removed as morphological and molecular vouchers, respectively, and the rest of the body was immersed in RNAlater (Sigma), crushed with a sterile pestle, and stored at −60°C until further processing. Specimens used for DNA extraction were immersed in 96% alcohol immediately after capture and stored at −20°C for later identification and processing (Additional file 5: Table S2). Vouchers are deposited at Beijing Forestry University, China.

All calyptrate families were sampled, i.e., all Hippoboscoidea (here following Pape et al. [45] in considering the Nycteribiidae and Streblidae to be subordinate to Hippoboscidae), muscoid grade, and Oestroidea. All currently recognized calliphorid subfamilies were sampled (Additional file 1: Table S1). *Drosophila melanogaster* was included to root the tree because of the growing evidence that Ephydroidea are the sister group to Calyptratae [35, 46–49]. Transcriptomic data were either generated for this study or downloaded from GenBank and other databases (Additional file 1: Table S1).

### Nucleic acids extraction, sequencing, and data processing

The total RNA was extracted using TRIzol (Invitrogen Life Technologies; Catalog # 15596-026), with the total RNA concentration and RNA integrity number (RIN) for each extraction assessed using an Agilent 2100 Bioanalyzer with the RNA 6000 Nano kit (Agilent Technologies, Santa Clara, CA; Catalog # 5067-1511). About 200 ng to 1 μg of total RNA was purified to construct a cDNA library for each sample using the TruSeq RNA Sample Prep Kit v2 (Illumina, San Diego, USA; Catalog # RS-122-2001) following the manufacturer’s instructions. An Illumina HiSeq 4000 sequencer was employed to generate paired-end reads for each library (Additional file 5: Table S2).

The total genomic DNA was extracted using a QIAGEN DNeasy® Blood and Tissue Kit (Qiagen, Hilden, Germany) following the manufacturer’s instructions. An Illumina NovaSeq 6000 sequencer was used for paired-end sequencing with insert size of 350 bp (Additional file 5: Table S2).

FastQC [50] was used to assess the quality of the generated raw data. The data were then trimmed using Trimmomatic [51] installed on Computerome (http://www.computerome.dtu.dk), with adapter sequences trimmed referring to the self-provided Illumina adapter sequence database. Also leading and trailing bases with quality below 30 were removed for each read, then each read was scanned with a 4-base wide sliding window to cut reads with the average quality below 15 within the window, and only reads with a minimum length of 36 bp were retained. Trimmomatic was used until the FastQC estimate of “per base sequence quality” was above 20, and no adapter sequences were detected in “overrepresented sequences” and “adapter content.”

Trinity (version 2.4.0) [52] was used to perform de novo assemblies for RNAseq data with default settings as described in Haas et al. [53]. After assembling, we estimated the average coverage of each transcript by mapping back the raw reads to assembled contigs using the *perl* script “align_and_estimate_abundance.pl” from Trinity. Only transcripts with average coverage above ten were kept. SOAPdenovo2 [54] was used for de novo assembling of genomic data with default settings. The assemblies were then trimmed for vector contamination referring UniVec Core database using Geneious (version 7.1.5) (Biomatters, Auckland, New Zealand). Only contigs with a length at least 200 bp were used from further analyses.

The data processing followed Misof et al. [36], Kutty et al. [19, 55], and Yan et al. [56]. We used orthograph [57] for reciprocal search to infer orthology for each target taxon following the workflow of Misof et al. [36] and Kutty et al. [55]. Orthograph (version 0.6.1) was run using reference ortholog sets [19, 56] with the alignment-program set as mafft-linsi, hmmbuild-program as hmmbuild, hmmsearch-program as hmmsearch, blast-program as blastp, exonerate-program as exonerate, blast-score-threshold as 10, and blast-evalue-threshold as 1e−05. After orthologous gene clusters for our assemblies were successfully assigned, the *perl* script summarize_orthograph_results.pl was used to summarize both NT and AA sequences of transcripts recognized as single-copy genes for all taxa. MAFFT (version 7.310) [58] with the L-INS-i algorithm was subsequently employed to construct MSA of all AA sequences. Outliers that were putatively misaligned were checked and re-aligned, and sequences were removed if they were still detected as outliers in the additional checking after refinement [19, 59]. For each dataset, all reference sequences except for *Drosophila melanogaster* were then removed from each multiple sequence alignment (MSA). PAL2NAL (version 14) [60] modified by Misof et al. [36] was used to align NT sequences with the above-refined amino acid MSAs as blueprints. Aliscore (version 2.2) [61–63] was used with default parameters to identify ambiguously or randomly aligned amino acid MSA sections of each orthologous gene, which were subsequently removed with ALICUT (version 2.3) [64]. The corresponding ambiguous sites of nucleotide MSAs were identified with custom *perl* scripts from Misof et al. [36] and removed with ALICUT (version 2.3). The MSAs were then recoded with leading and trailing gaps replaced with “N” for NT sequences and “X” for AA sequences. Subsequently, the amino acid MSAs for each dataset were concatenated into supermatrix using FASconCAT-G [65]. MARE (version 0.1.2-rc) [66] was then used to improve the overall information content of the matrix, with the flag “-c” used to keep all taxa with fewer genes that would otherwise be removed. The corresponding nucleotide supermatrix with improved information content was built using FASconCAT-G.

At the onset of the study, we noticed the different phylogenetic positions of Chrysomyinae between our study (Fig. 3) and that of Kutty et al. [19]. There are two main differences between these two studies, i.e., taxon sampling and ortholog reference. We therefore performed reconstructions with different taxon representations and ortholog reference sets to interrogate the contrasting placements of Chrysomyinae. We have two taxon sets: a larger one with 39 species (Ltax) and a smaller (reduced) one with 26 species (Stax). The data were analyzed using two ortholog sets, the Diptera ortholog reference (Dref) with ortholog set of 3755 single-copy nuclear protein-encoding genes recognized from official gene sets of five dipteran species (*Aedes aegypti*, *Drosophila melanogaster*, *Glossina morsitans*, *Lucilia cuprina*, *Musca domestica*) (Additional file 6: Table S3) using OrthoFinder (version 1.1.10) [67], and the Antliophora ortholog set (Aref; recognized from official gene sets of *Tribolium castaneum*, *Mayetiola destructor*, *Bombyx mori*, *Anopheles gambiae*, *Drosophila melanogaster*) with a slightly smaller number of single-copy nuclear protein-encoding genes (3288) used in Kutty et al. [19]. Following the process described above, our analysis of the data started with Ltax being analyzed with Dref as the ortholog set (Dref_Ltax; 2221 genes) and then based on Ltax with Aref (Aref_Ltax; 1764 genes) followed by Stax with Dref (Dref_Stax; 2003 genes) and Stax with Aref (Aref_Stax; 1465 genes), respectively. Amino acid and nucleotide sequences of transcripts recognized as single-copy genes were used to generate four datasets (Table 1). Matrices of amino acid (AA), 2nd-codon positions (NT2), and 1st & 2nd-codon positions (NT12) of each dataset were generated. AliStat (version 1.7) [68], MARE, and Symtest (version 2.0.47) [69] were used to report alignment diagnostics of each supermatrix, e.g., site coverage of the matrices, and to explore whether or not the matrices matched conditions assumed by most models including stationarity, reversibility, and homogeneity (Additional file 2: Figs. S3–S6). The AA matrix for each dataset was also recorded as a six-state Dayhoff group using the “pgrecodeseq” command in the PHYLOGEARS v.2.0 tool package [70] for parsimony tree construction.

### Phylogenetic inference and topology test

#### Concatenation-based reconstruction

ML trees were inferred using IQ-TREE (version 1.6.8) [71] based on AA, NT2, and NT12, and MP trees inferred using TNT [72] for AA matrices of all four datasets (Table 1) yielding 16 concatenation-based phylogeny reconstructions.

IQ-TREE (version 1.6.8) [71] was used for ML reconstruction, with the best model for each gene estimated by the self-implemented ModelFinder [73] following the Akaike Information Corrected Criterion (AICc) score [74], and branch support estimated with 100 standard bootstrap resampling analysis.

The MP tree was constructed using six-state Dayhoff recoded matrices. TNT (version 1.5) [72] was run with new technology searches, level 10, hits 20, gaps coded as missing data, and node support assessed by jackknife resampling with 1000 replicates at 36% deletion following Kutty et al. [19].

#### Coalescent-based reconstruction

The coalescent-based phylogeny was conducted only using the AA alignments of Dref_Ltax, because this dataset has the highest number of genes. Amino acids of all 2221 MSAs were used to construct gene trees using IQ-TREE, respectively, with the best model for each MSA estimated by ModelFinder based on AICc and branch support derived from 100 standard bootstrap replicates. All the constructed gene trees were subsequently used to infer a coalescent-based phylogeny using ASTRAL (version 5.6.1) with default parameters [75].

#### Tests for Chrysomyinae placement

The Chrysomyinae had different placements across the phylogenetic analyses of various datasets. They were either placed together with Phumosiinae as the sister group of most other calliphorids (i.e., T1 in Fig. 5A; (group 2a, (group 2b, group 3)) in Fig. 6A), as sister group to Luciliinae, Aphyssurinae-Calliphorinae-Melanomyinae-Toxotarsinae (i.e., T2 in Fig. 5A; group 3 in Fig. 6A), or in fewer cases as sister group to Rhiniidae and Bengaliinae (i.e., T3 in Fig. 5A; group 2B). We therefore performed a modified FcLM [76], partition log-likelihood analyses [77], and an approximately unbiased (AU) test [78] using amino acid alignments of the dataset with the largest number of genes (i.e., Dref_Ltax) to compare the amount of phylogenetic signal for competing hypotheses.

The group definitions for FcLM were as depicted in Fig. 6A. The paraphyly of group 2 means that FcLM is not applicable directly to our dataset. Therefore, we modified the analyses and performed FcLM with two variations after splitting this group into group 2A and group 2B, which were both inferred as monophyletic but never recovered as sister groups. For one analysis, we pruned data of group 2B, keeping the remaining four clusters in Fig. 6A, and performed FcLM as implemented in IQ-TREE. For the other analysis, we pruned group 2A and kept group 2B.

To perform the partitioned log-likelihood analysis, we calculated ΔpL_i_ of each partition by subtracting the likelihood for T3 (Δ_T3_pL_i_) or T2 (Δ_T2_pL_i_) of each gene from the corresponding likelihood for T1 (Δ_T1_pL_i_) following [77, 79] (i.e., ΔpL_i_ = Δ_T1_pL_i_ − Δ_T3_pL_i_, or ΔpL_i_ = Δ_T1_pL_i_ − Δ_T2_pL_i_), and values of ΔpL_i_ were then plotted for visualization. The same calculation and plotting were performed for T1 and T2. The AU test implemented in IQ-TREE was conducted for topologies T1, T2, and T3 for 10,000 replicates, respectively.

### Reconstruction of ancestral state of adult metallic color

Character states of terminal taxon were collected from the literature [7, 8, 12, 80], as shown in Table S4 (Additional file 7). Metallic color was coded as present or absent according to the coloration measured by the eye, and no attempt was made to present a multistate coding because of the complexity involved in transitions between different states. Species with bodies that were partially metallic were therefore coded as the metallic color present, e.g., for Mesembrinellidae, where the included species has only abdominal metallic coloration. Character states were treated with equal weight because of the impossible measurement of transition among different states. Bayesian binary Markov chain Monte-Carlo (BBM) [81] implemented in RASP [82] with default settings as described in Yan et al. [56, 83] was run to reconstruct the ancestral state.

## Supplementary Information

**Additional file 1: Table S1.** Taxon sampling [19, 56, 84–88] in this study and statistics of analyses.

**Additional file 2: Figure S1.** Maximum Likelihood tree inferred from the amino acid matrix of dataset Dref_Ltax. **Figure S2.** Bayesian reconstructions of ancestral states of adult metallic color. **Figure S3.** Heatmaps showing data coverage and homogeneity test of matrices Dref_Ltax. **Figure S4.** Heatmaps showing data coverage and homogeneity test of matrices Dref_Stax. **Figure S5.** Heatmaps showing data coverage and homogeneity test of matrices Aref_Ltax. **Figure S6.** Heatmaps showing data coverage and homogeneity test of matrices Aref_Stax.

**Additional file 3.** Score differences of partition log-likelihood for each locus recorded between the alternative hypotheses, T1 and T2 and between T1 and T3.

**Additional file 4.** Ancestral construction of adult metallic color of blowflies.

**Additional file 5: Table S2.** Collecting and sequencing information of newly sequenced species.

**Additional file 6: Table S3.** Genomes used to prepare dipteran orthologous references.

**Additional file 7: Table S4.** Character states and coding of each terminal taxon for ancestral construction.

## Data Availability

All data related to this publication are submitted to the GenBank databases under BioProject accession number PRJNA611871 [84].
