# Supplementary material for: Monophyletic blowflies revealed by phylogenomics
Source: BMC Biol. 2021 Oct 27;19:230. doi: 10.1186/s12915-021-01156-4 (PMC8555136; doi:10.1186/s12915-021-01156-4)
Supplement: Supplementary file 1 — Additional file 1: Table S1. Taxon sampling [19, 56, 84–88] in this study and statistics of analyses. [file 12915_2021_1156_MOESM1_ESM.pdf]

Monophyletic blowflies revealed by phylogenomics

Liping Yan, Thomas Pape, Karen Meusemann, Sujatha Narayanan Kutty, Rudolf Meier, Keith M. Bayless, Dong Zhang

Additional file 1: Table S1. Taxon sampling for phylogeny construction in the present study. Species with names in bold represent newly generated data.

Superfamily	Family	Subfamily	Species	Accession / version and reference	Number of assembled contigs	Dipteran orthologous groups in full dataset
	Drosophilidae		*Drosophila melanogaster* †	FB2013_03	N.A. §	3755
Hippoboscoidea	Hippoboscidae		*Ortholfersia macleayi* †	SRR1695374	59827	3345
	Glossinidae		*Glossina morsitans*	GmorY1.6	N.A. §	3755
Muscoid grade	Anthomyiidae		*Eustalomyia vittipes* †	SRR1695308	14303	2475
	Fanniidae		*Fannia canicularis* †	SRR8236841	51836	2778
	Muscidae	Azeliinae	*Muscina stabulans* †	SRR6724172	51691	3089
		Muscinae	*Musca domestica* †	MdomA1.3	N.A. §	3755
	Scathophagidae	Scathophaginae	*Scathophaga stercoraria* †	SRR8236843	39976	2014
Oestroidea	Calliphoridae	Ameniinae	*Amenia* sp. †	SRR6724164	41939	2912
			Silbomyia hoeneana	SRR11434663	169143	3175
		Aphyssurinae	***Aphyssura* sp.**	SRR13924097	421872	1787
		Bengaliinae	***Bengalia* sp.**	SRR11434662	72825	2876
			Verticia nigra †	SRR6724159	82170	3327
		Calliphorinae	*Calliphora vomitoria* †	SRR1695328	26705	3222
			***Polleniopsis* sp.**	SRR11434659	202652	3287
		Chrysomyinae	*Chrysomya rufifacies* †	SRR500993	183402	3578
			Chrysomya megacephala	SRR620248	78890	3013
			Cochliomyia hominivorax	SRR1532687	26600	2020
			***Protocalliphora* sp.**	SRR11434661	115669	3105

Superfamily	Family	Subfamily	Species	Accession / version and reference	Number of assembled contigs	Dipteran orthologous groups in full dataset
			Protophormia terraenovae	DRR087979	199281	3438
		Helicoboscinae	*Eurychaeta muscaria* †	SRR6724165	83849	3322
		Luciliinae	***Hypopygiopsis tumrasvini***	SRR11434660	68271	2550
			Lucilia cuprina †,‡	GCA_001187945.1	N.A. §	3755
		Melanomyinae	***Melinda viridicyanea***	SRR11434658	86702	3139
		Phumosiinae	***Phumosia chukanella***	SRR11434657	136747	3368
		Toxotarsinae	***Sarconesia magellanica***	SRR13924098	275286	3683
	Mesembrinellidae		*Mesembrinella bellardiana* †	SRR6724170	26792	2336
	Polleniidae		*Pollenia* sp. †	SRR8236845	104465	3137
	Mystacinobiidae		*Mystacinobia zelandica* †	SRR6724158	59619	3267
	Oestridae	Cuterebrinae	*Cuterebra austeni* †	SRR1695306	9936	1686
	Rhiniidae		*Stomorhina subapicalis* †	SRR1695394	29906	3214
	Rhinophoridae		*Bixinia* sp. †	SRR6724163	37044	2827
			Stevenia sp. †	SRR6724161	70211	3292
	Sarcophagidae	Miltogrammatinae	*Miltogramma oestraceum* †	SRR10753909	48023	2427
		Paramacronychiinae	*Agria mihalyii* †	SRR10753923	193051	3310
		Sarcophaginae	*Sarcophaga carnaria* †	SRR10753913	68497	3001
	Tachinidae	Phasiinae	*Gymnosoma nitens* †	SRR6724168	69801	3412
		Exoristinae	*Pseudogonia rufifrons* †	SRR6724155	81968	3369
	Ulurumyiidae		*Ulurumyia macalpinei* †	SRR6724160	47265	2957

†- Data used in the analysis of smaller sampling (Stax).

‡- This taxon was changed into *Lucilia sericata* (Access: SRR350021) in the analysis of smaller sampling (Stax).

§- These genomes were downloaded as assembled and annotated, hence assembly information is not available.
