# Supplementary material for: Monophyletic blowflies revealed by phylogenomics
Source: BMC Biol. 2021 Oct 27;19:230. doi: 10.1186/s12915-021-01156-4 (PMC8555136; doi:10.1186/s12915-021-01156-4)
Supplement: Supplementary file 2 — Additional file 2: Figure S1. Maximum Likelihood tree inferred from the amino acid matrix of dataset Dref_Ltax. Figure S2. Bayesian reconstructions of ancestral states of adult metallic color. Figure S3. Heatmaps showing data coverage and homogeneity test of matrices Dref_Ltax. Figure S4. Heatmaps showing data coverage and homogeneity test of matrices Dref_Stax. Figure S5. Heatmaps showing data coverage and homogeneity test of matrices Aref_Ltax. Figure S6. Heatmaps showing data coverage and homogeneity test of matrices Aref_Stax. [file 12915_2021_1156_MOESM2_ESM.pdf]

Monophyletic blowflies revealed by phylogenomics

Liping Yan, Thomas Pape, Karen Meusemann, Sujatha Narayanan Kutty, Rudolf Meier, Keith M. Bayless, Dong Zhang

Additional file 2. Figures S1-S6

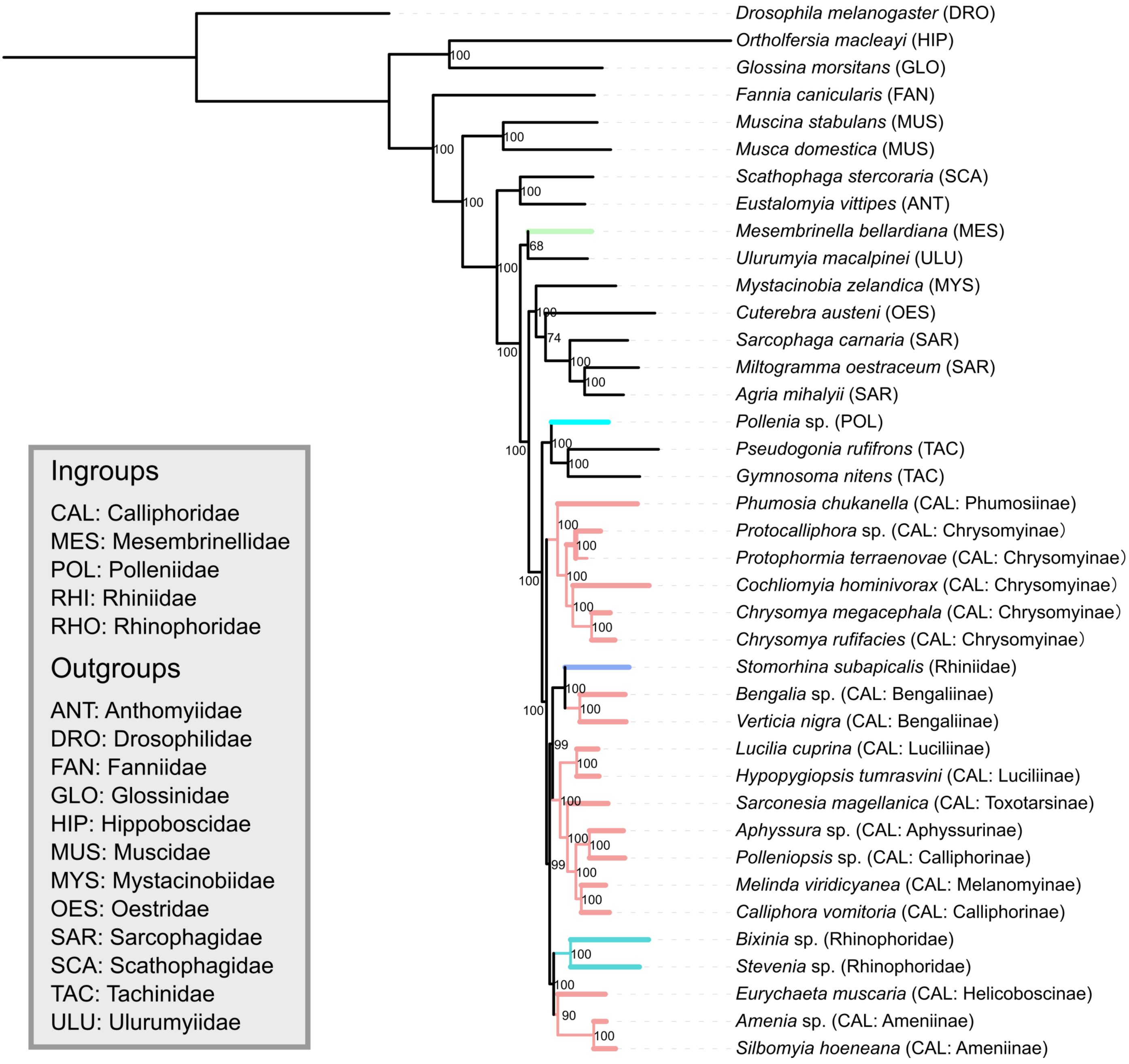

Fig. S1. Maximum Likelihood (ML) tree inferred from the amino acid matrix of dataset Dref_Ltax, with support values of ML bootstrap (MLBS) presented beside nodes.

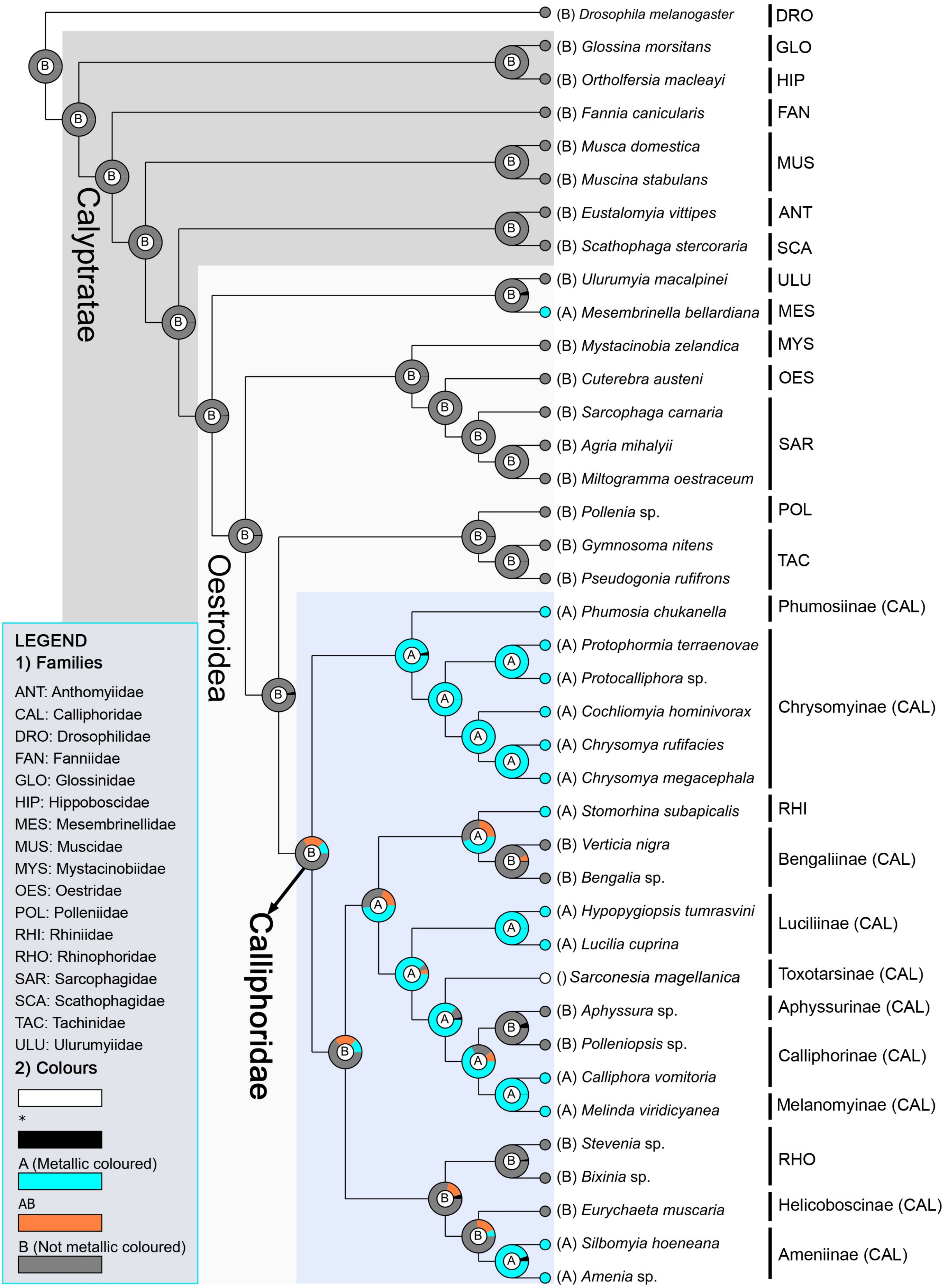

Fig. S2. Bayesian reconstructions of ancestral states of adult metallic color. Probabilities are shown as color proportions for each node.

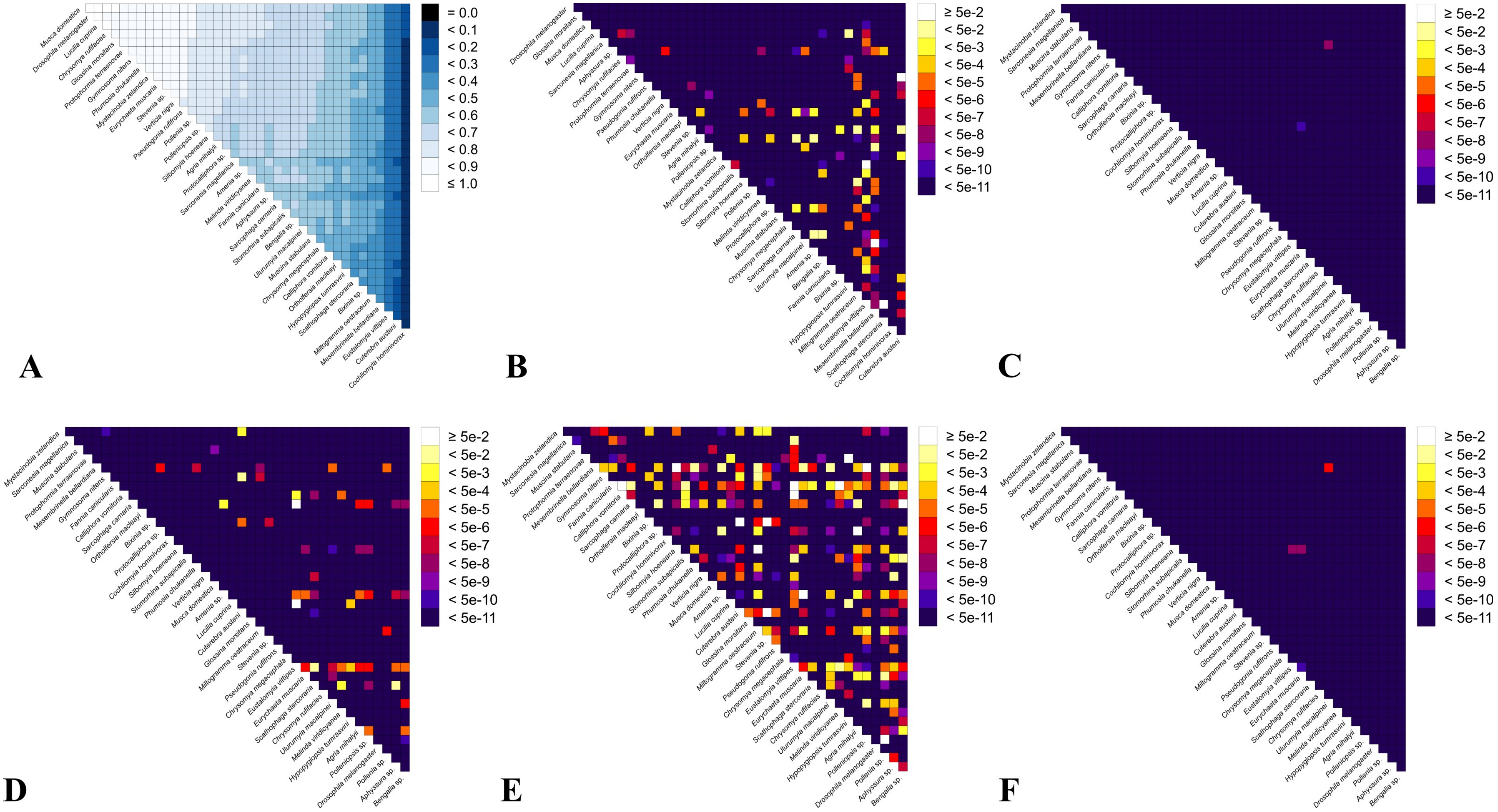

Fig. S3. Heatmaps showing data coverage and homogeneity test of matrices Dref_Ltax. A – Data coverage estimated by Alistat test; B – homogeneity of amino acid matrix estimated by symtest; C – homogeneity of all three codon positions of nucleotide matrix estimated by symtest; D – homogeneity of 1st-codon position of nucleotide matrix estimated by symtest; E – homogeneity of 2nd-codon position of nucleotide matrix estimated by symtest; F – homogeneity of 3rd-codon position of nucleotide matrix estimated by symtest.

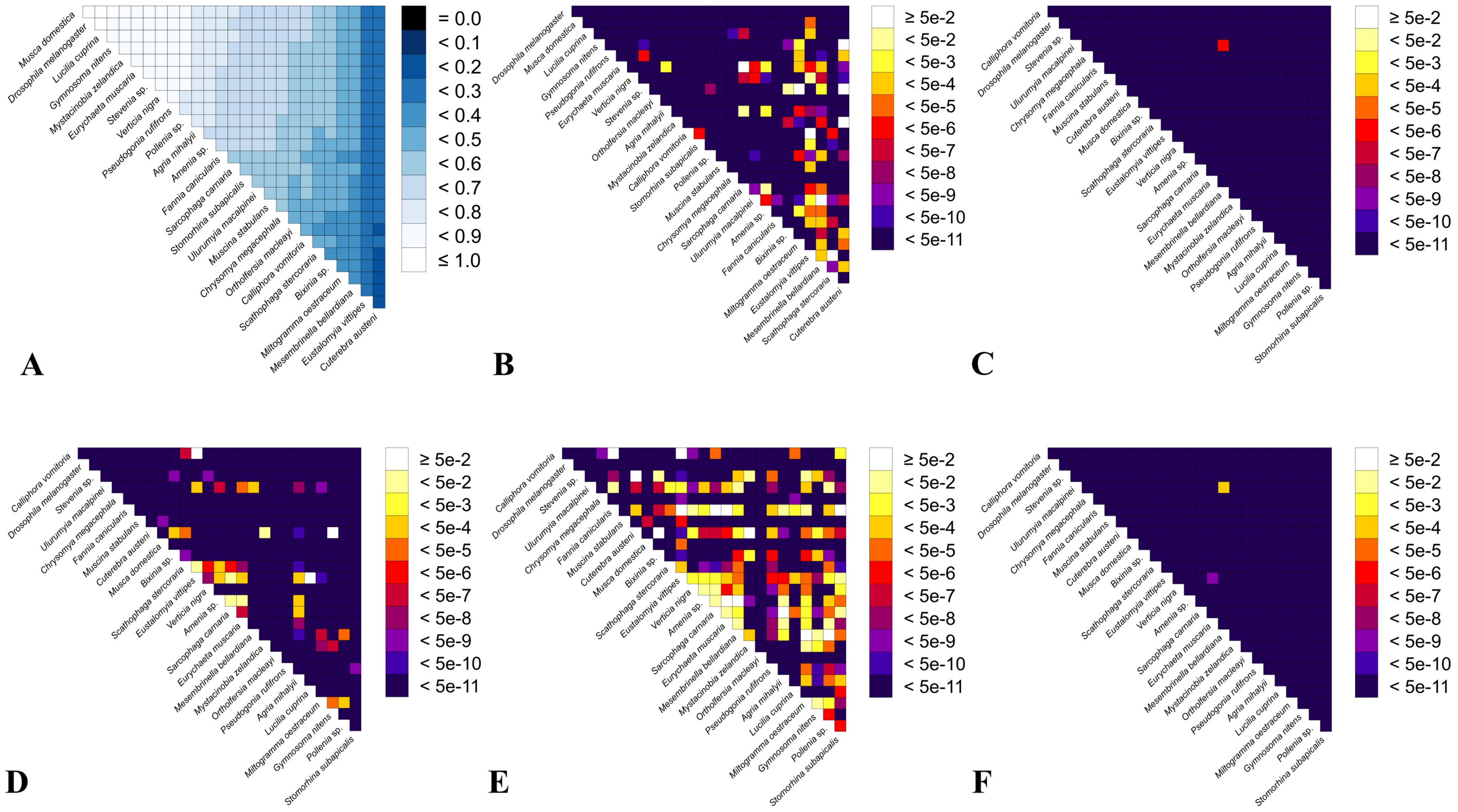

Fig. S4. Heatmaps showing data coverage and homogeneity test of matrices Dref_Stax. A – Data coverage estimated by Alistat test; B – homogeneity of amino acid matrix estimated by symtest; C – homogeneity of all three codon positions of nucleotide matrix estimated by symtest; D – homogeneity of 1st-codon position of nucleotide matrix estimated by symtest; E – homogeneity of 2nd-codon position of nucleotide matrix estimated by symtest; F – homogeneity of 3rd -codon position of nucleotide matrix estimated by symtest.

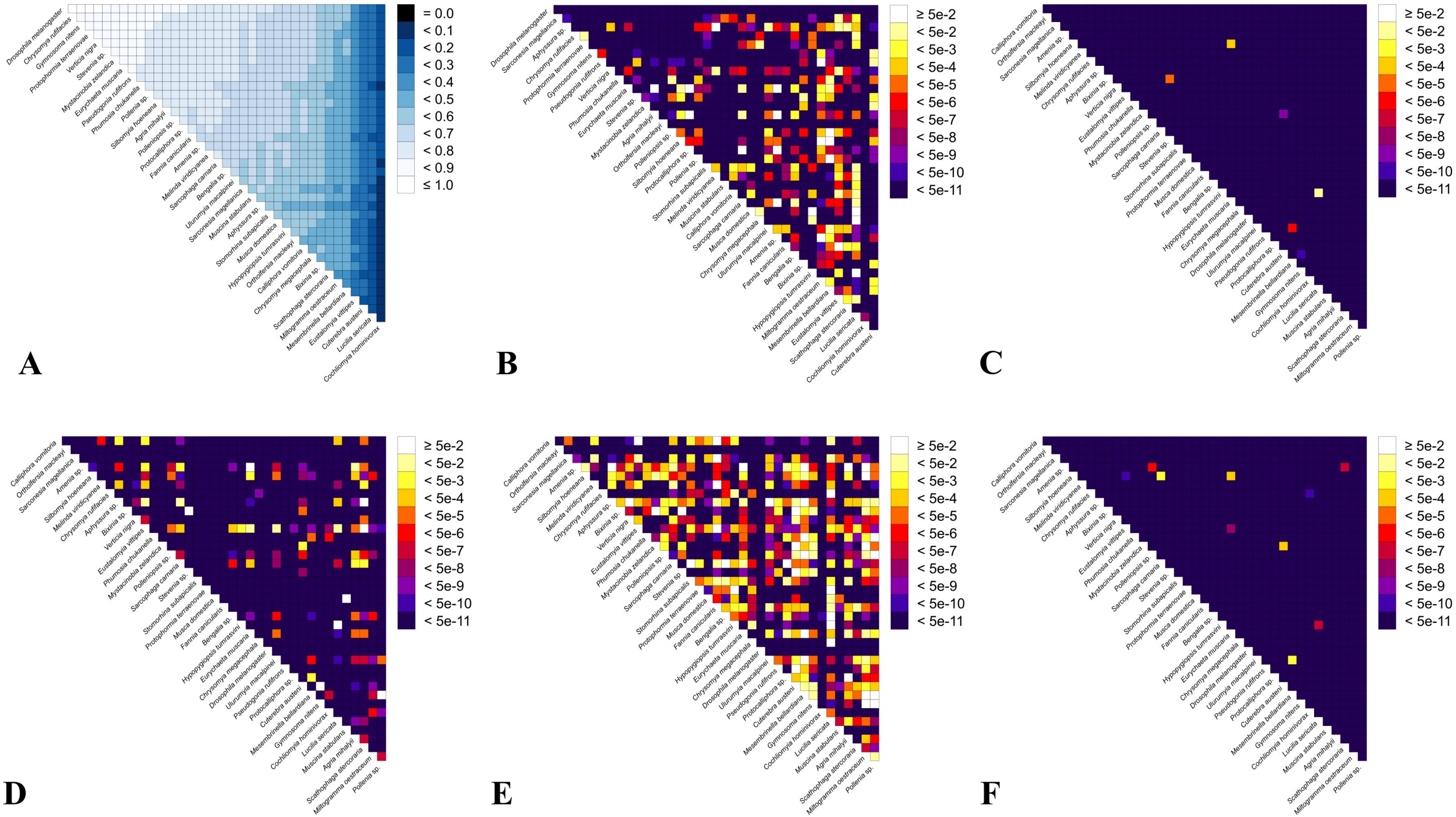

Fig. S5. Heatmaps showing data coverage and homogeneity test of matrices Aref_Ltax. A – Data coverage estimated by Alistat test; B – homogeneity of amino acid matrix estimated by symtest; C – homogeneity of all three codon positions of nucleotide matrix estimated by symtest; D – homogeneity of 1st-codon position of nucleotide matrix estimated by symtest; E – homogeneity of 2nd-codon position of nucleotide matrix estimated by symtest; F – homogeneity of 3rd -codon position of nucleotide matrix estimated by symtest.

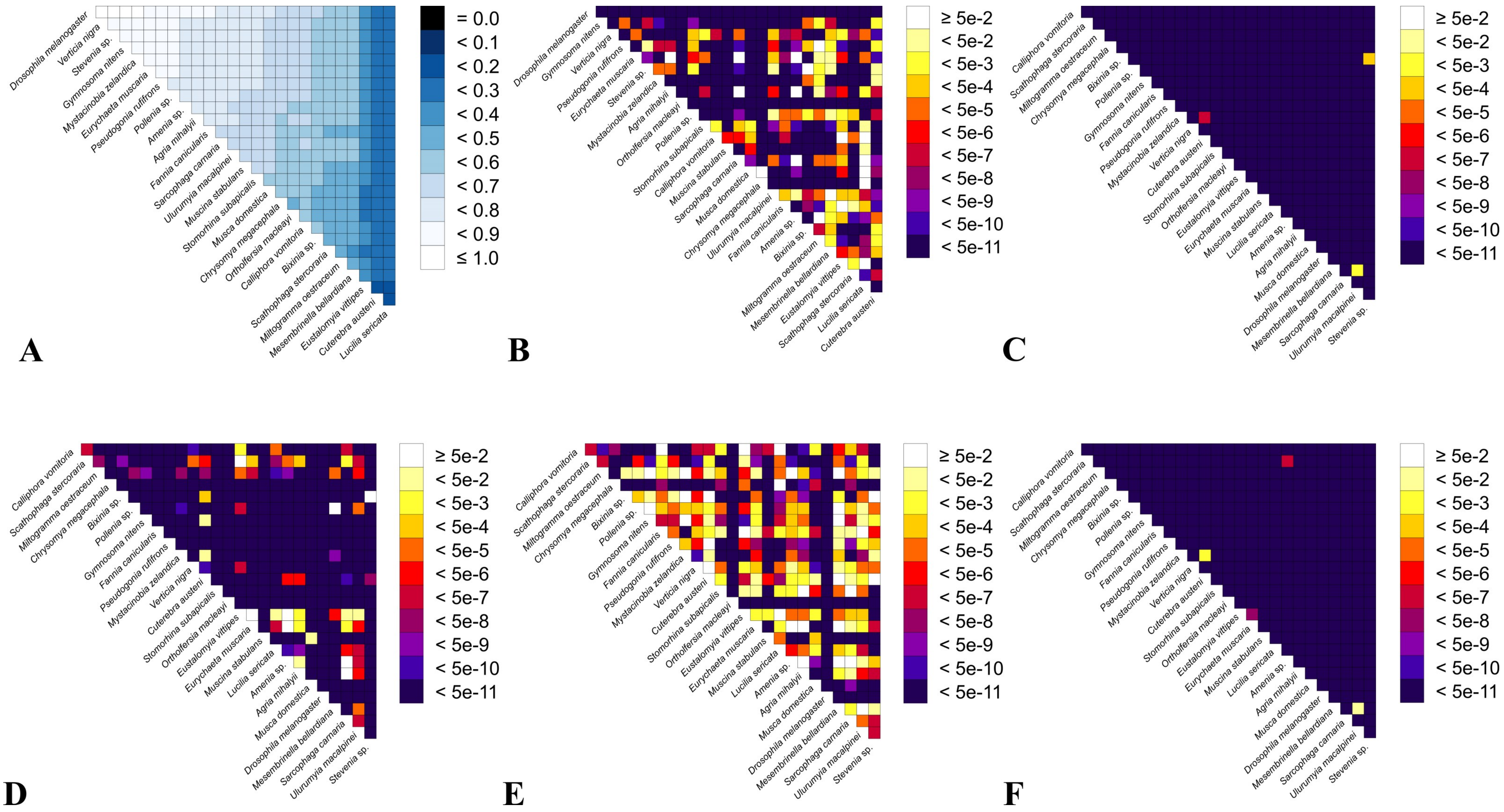

Fig. S6. Heatmaps showing data coverage and homogeneity test of matrices Aref_Stax. A – Data coverage estimated by Alistat test; B – homogeneity of amino acid matrix estimated by symtest; C – homogeneity of all three codon positions of nucleotide matrix estimated by symtest; D – homogeneity of 1st-codon position of nucleotide matrix estimated by symtest; E – homogeneity of 2nd-codon position of nucleotide matrix estimated by symtest; F – homogeneity of 3rd-codon position of nucleotide matrix estimated by symtest.
