# Supplementary material for: Monophyletic blowflies revealed by phylogenomics
Source: BMC Biol. 2021 Oct 27;19:230. doi: 10.1186/s12915-021-01156-4 (PMC8555136; doi:10.1186/s12915-021-01156-4)
Supplement: Supplementary file 5 — Additional file 5: Table S2. Collecting and sequencing information of newly sequenced species. [file 12915_2021_1156_MOESM5_ESM.pdf]

Monophyletic blowflies revealed by phylogenomics

Liping Yan, Thomas Pape, Karen Meusemann, Sujatha Narayanan Kutty, Rudolf Meier, Keith M. Bayless, Dong Zhang

Additional file 5: Table S2. Collecting and sequencing information of calliphorid phylogenomic data documented in the present study.

Species	Collecting Location	Collection date	Developmental Stage	Identified by	RNA Extraction [total amount (μg) /RIN]	Library ID	Read Length/bp
Aphyssura sp.	Australia		Adult	K. Bayless	Not Available	FKDN202339100-1A	150
Bengalia sp.	Wenshan, Yunnan, China	August, 2015	Adult	T. Pape	19.9 / 6.3	WHINSrohEAABRAAPEI-20	100
Hypopygiopsis tumrasvini	Jianfengling, Hainan, China	June, 2016	Adult	T. Pape	3.06 / 6.2	WHINSvsjEAAARAAPEI-72	150
Melinda viridicyanea	North East Zealand Lejre, Denmark	August, 2015	Adult	T. Pape	14.76 / 6.3	WHINSrohEAACRAAPEI-21	100
Phumosia chukanella	Morogoro region Udzungwa Mts Natl Park, Tanzania	July, 2019	Adult	T. Pape	12.10 / 7	FRAS190118440-1a	150
Polleniopsis sp.	Jianfengling, Hainan, China	June, 2016	Adult	T. Pape	6.75 / 5.5	WHINSmabEAABRAAPEI-76	150
Protocalliphora sp.	Songshan, Beijing, Chinae	June, 2016	Adult	D. Zhang, T. Pape	65.6 / 5.9	WHINSmabEAAARAAPEI-75	150
Sarconesia magellanica	Azuay, Ecuador	December, 2017	Adult	T. Pape	Not Available	BKDN200012960-1A	150
Silbomyia hoeneana	Jianfengling, Hainan, China	June, 2016	Adult	T. Pape	9.6 / 5.1	WHINSmabEAAJRAAPEI-84	150
