# Supplementary material for: Monophyletic blowflies revealed by phylogenomics
Source: BMC Biol. 2021 Oct 27;19:230. doi: 10.1186/s12915-021-01156-4 (PMC8555136; doi:10.1186/s12915-021-01156-4)
Supplement: Supplementary file 6 — Additional file 6: Table S3. Genomes used to prepare dipteran orthologous references. [file 12915_2021_1156_MOESM6_ESM.pdf]

Monophyletic blowflies revealed by phylogenomics

Liping Yan, Thomas Pape, Karen Meusemann, Sujatha Narayanan Kutty, Rudolf Meier, Keith M. Bayless, Dong Zhang

Additional file 6: Table S3. Genomic data used to prepare dipteran orthologous references.

Family	Species	Gene set/ Assembly	Downloaded from
Culicidae	*Aedes aegypti* (Linnaeus)	AaegL3.4	https://www.vectorbase.org/organisms/aedes-aegypti
Drosophilidae	*Drosophila melanogaster* Meigen	5.51	http://www.flybase.org/reports/FBgn0015805
Glossinidae	*Glossina morsitans* Westwood	GmorY1.6	https://www.vectorbase.org/organisms/glossina-morsitans
Calliphoridae	*Lucilia cuprina* Wiedemann	GCA_001187945.1	http://metazoa.ensembl.org/Lucilia_cuprina/Info/Annotation/
Muscidae	*Musca domestica* Linnaeus	MdomA1.3	https://www.vectorbase.org/organisms/musca-domestica
