# Supplementary material for: Monophyletic blowflies revealed by phylogenomics
Source: BMC Biol. 2021 Oct 27;19:230. doi: 10.1186/s12915-021-01156-4 (PMC8555136; doi:10.1186/s12915-021-01156-4)
Supplement: Supplementary file 7 — Additional file 7: Table S4. Character states and coding of each terminal taxon for ancestral construction. [file 12915_2021_1156_MOESM7_ESM.pdf]

Monophyletic blowflies revealed by phylogenomics

Liping Yan, Thomas Pape, Karen Meusemann, Sujatha Narayanan Kutty, Rudolf Meier, Keith M. Bayless, Dong Zhang

Additional file 7: Table S4. Character and coding of each taxon.

Family	Species	Metallic color
Anthomyiidae	*Eustalomyia vittipes*	No
Calliphoridae	*Amenia* sp.	Yes
	Aphyssura sp.	No
	Bengalia sp.	No
	Calliphora vomitoria	Yes
	Chrysomya megacephala	Yes
	Chrysomya rufifacies	Yes
	Cochliomyia hominivorax	Yes
	Eurychaeta muscaria	No
	Hypopygiopsis tumrasvini	Yes
	Lucilia cuprina	Yes
	Melinda viridicyanea	Yes
	Phumosia chukanella	Yes
	Polleniopsis sp.	No
	Protocalliphora sp.	Yes
	Protophormia terraenovae	Yes
	Sarconesia magellanica	Not available
	Silbomyia hoeneana	Yes
	Verticia nigra	No
Drosophilidae	*Drosophila melanogaster*	No
Fanniidae	*Fannia canicularis*	No
Glossinidae	*Glossina morsitans*	No
Hippoboscidae	*Ortholfersia macleayi*	No
Muscidae	*Musca domestica*	No
	Muscina stabulans	No
Mesembrinellidae	*Mesembrinella bellardiana*	Yes
Mystacinobiidae	*Mystacinobia zelandica*	No
Oestridae	*Cuterebra austeni*	No
Polleniidae	*Pollenia* sp.	No
Rhiniidae	*Stomorhina subapicalis*	Yes
Rhinophoridae	*Stevenia* sp.	No
	Bixinia sp.	No
Sarcophagidae	*Sarcophaga carnaria*	No
	Agria mihalyii	No
	Miltogramma oestraceum	No
Scathophagidae	*Scathophaga stercoraria*	No
Tachinidae	*Gymnosoma nitens*	No
	Pseudogonia rufifrons	No
Ulurumyiidae	*Ulurumyia macalpinei*	No
